# Surgical outcomes of adjustment on recessed lateral rectus muscle versus resected medial rectus muscle for intermittent exotropia

**DOI:** 10.1186/s12886-022-02437-4

**Published:** 2022-05-25

**Authors:** Donghun Lee, So Hyung Lee, Sook Young Kim

**Affiliations:** Department of Ophthalmology, Daegu Catholic University School of Medicine, 33 Duryugonwon-ro 17 gil, Nam-gu, 42472 Daegu, Korea

**Keywords:** Adjustable strabismus surgery, Intermittent exotropia, Lateral rectus recession, Recess-resect

## Abstract

**Background:**

To evaluate the effectiveness of resected muscle adjustment compared with the recessed muscle adjustment in patients with intermittent exotropia.

**Methods:**

This retrospective clinical investigation analyzed the data of patients who underwent strabismus surgery with adjustment. Patients who were followed-up for at least one and half year after adjustment were enrolled. They were divided into two groups; patients who underwent adjustment on recessed lateral rectus muscle (LR-Adj group) and adjustment on resected medial rectus muscle (MR-Adj group). Postoperative changes were compared. Surgical success was defined as horizontal deviation < 5 prism diopters (PD) esodeviation and < 10 PD exodeviation on distance measurement at 1.5 years postoperatively.

**Results:**

Forty patients were included; LR-Adj group included 21 and MR-Adj group included 19 patients. The mean esodeviation at distance fixation immediately after adjustment was 8.1 ± 5.4 PD in the LR-Adj group and 8.4 ± 4.7 PD in the MR-Adj group (*P* = 0.843). Postoperative exodrift occurred in both groups, and amount of exodeviation after 1.5 year were not significantly different. For the comparison of the amount of exodrift at near measurement, the amount of exodrift within 1 month after surgery was smaller in the MR-Adj group than that in the LR-Adj group (*P* = 0.01). Surgical success rates were 81.0% in the LR-Adj group and 84.2% in the MR-Adj group (*P* = 0.559).

**Conclusions:**

The smaller amount of exodrift in the MR-Adj group may mean that the positional stability of the resected muscle is favorable in the early post-adjustment period. However, there was no significant difference groups in the final exodeviation and surgical success rate between the groups. Resected muscle adjustment was as effective as the conventional recessed muscle adjustment.

## Background

The adjustment surgery, first described by Bielschowsky in 1907 [1] and popularized by Jampolsky in the 1970s [2], has been used whenever the surgical outcome is unpredictable. Indications for this technique may vary, and include restrictive, paralytic, and long-standing complex strabismus [3–5]. This technique is often used for large angle cases or reoperation in patients with intermittent exotropia; however, it can also be an option for all patients willing to undergo the procedure [6].

As reported by Wright [7], it is recognized that the adjustment surgery is generally used for rectus muscle recessions, and many previous reports describing adjustment have addressed adjustments to the recessed muscle [3–5]. Meanwhile, others have suggested that the adjustment surgery is adequate for resected muscle in horizontal strabismus [8, 9], and believe that resection using adjustable suture is superior in terms of stability.

To our knowledge, no previous studies have compared post-adjustment changes between resected and recessed muscles. For clinicians, the decision regarding which horizontal muscle to perform the adjustment surgery on is as important as deciding whether to perform it at all. The present study aimed to compare the effectiveness and surgical outcomes of recessed muscle and resected muscle adjustment in patients with intermittent exotropia.

## Methods

### Study design and participants

The Institutional Review Board (IRB) for Human Studies at Daegu Catholic University Medical Center (Daegu, Republic of Korea) reviewed and approved the protocol used in this study (IRB no. CR-20–195). Informed consent was obtained from all patients or their parent in the case of children < 16 years of age (retrospective study). All procedures adhered to the tenets of the Declaration of Helsinki. Data from patients with intermittent exotropia, who underwent strabismus surgery involving the adjustment surgery between January 2005 and August 2019 and followed-up for at least 1.5 years after surgery, were retrospectively analyzed. Only patients with the basic type of intermittent exotropia, with distance deviation within 10 diopters (D) of the near deviation, were included. Patients with other types of strabismus such as paralytic, restrictive, and sensory—were excluded from this study to eliminate other factors that may affect postoperative exodrift. In addition, patients who underwent previous extraocular muscle surgery and concurrent oblique muscle surgery were also excluded. Patients with A-V pattern strabismus, lateral incomitance, nystagmus or vertical deviation were also excluded. Furthermore, this study only included patients who underwent the adjustable suture procedure during strabismus surgery and adjustments the day after surgery. Those who did not require adjustment the next day and simply underwent tying off were excluded.

### Surgical techniques

**Fig. 1:**
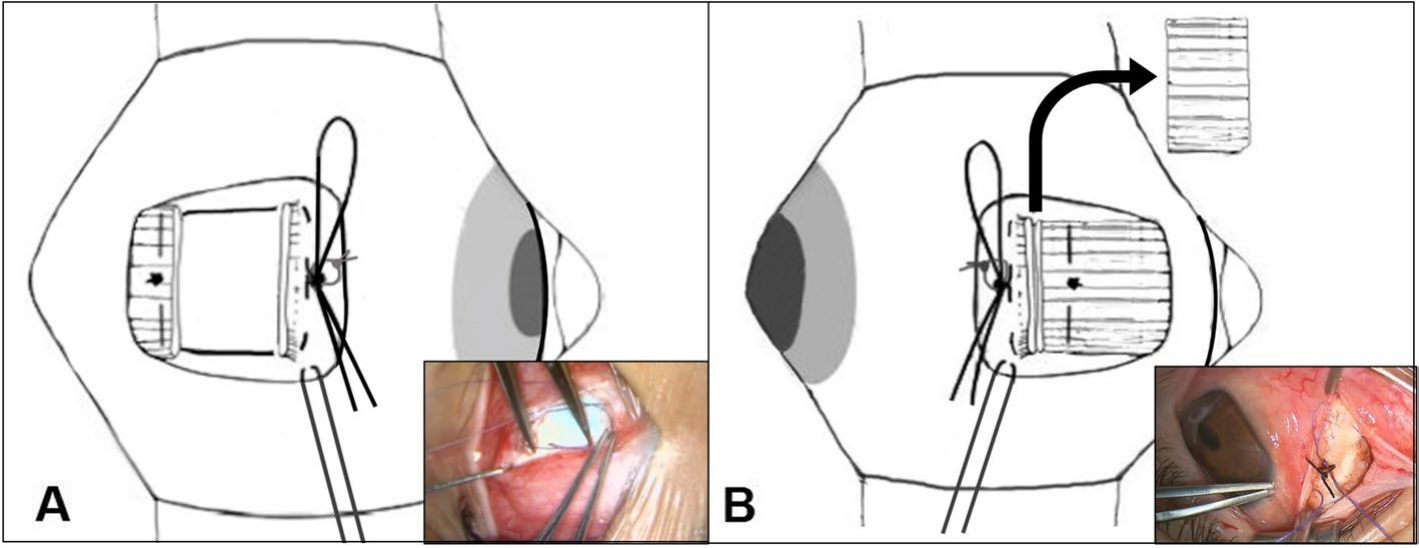
Adjustable suture techniques for the recessed muscle and the resected muscle. **A** The lateral rectus muscle on the right eye was recessed using hang back technique and was secured to the scleral insertion by a single loop bow-tie. **B** The resected medial rectus muscle on the right eye was suspended 2 mm posterior to the insertion site and was secured using a single loop bow-tie suture. This provided the space for advancing during the adjustment procedure if needed. In both surgeries, a bolster suture below the bow-tie suture and a traction suture near the insertion site were made

All strabismus surgeries were performed by one surgeon (SYK) using the fornix approach with the patients under general anesthesia. The surgical formula used for both groups, based on the largest angle ever measured, is presented in Table 1. Type of surgery, such as bilateral lateral rectus recession, unilateral lateral rectus recession, and medial rectus resection, depended on the amount of exodeviation and the presence of dominant eye. In the case of lateral rectus muscle recession with an adjustable suture (Fig. 1A), the muscle was disinserted from its insertion and secured using a 6–0 Vicryl suture. Spatula needles from the double-arm suture were passed perpendicular and then parallel to the scleral insertion line. The perpendicular scleral sutures were placed at this and the superior and inferior points of the insertion and then passed parallel toward the center of the insertion so that they met in the middle. After measuring the amount of recession using a caliper, the lateral rectus muscle was hung back posteriorly and was secured to the scleral insertion using a single-loop bowtie. A 4–0 Vicryl bolster suture was made below the bowtie suture to untie it easily during the adjustment procedure. Furthermore, a 6–0 Vicryl suture was placed intrasclerally near the insertion site as a traction suture to provide adequate exposure during the adjustment procedure. After placing all sutures under the conjunctiva, the conjunctiva was moved toward the fornix incision.

**Table 1:** Surgical table for intermittent exotropia

Deviation (PD)	Bilateral LR Rec (mm)	Unilateral R and R (mm)	Unilateral R and R with LR rec on the other eye (mm)
18 – 20	6	6/5	-
25	6.5	7/5	-
30	7	7/6	-
35	8	7.5/6	-
40	8.5	8/6.5	-
45	9	8.5/6.5	-
50	-	9/7	-
55	-	-	7/6, 8
60	-	-	7/6, 9
65	-	-	8/7, 9

*PD* Prism diopters, *LR* Lateral rectus, *R and R* Lateral rectus recession and medial rectus resection, *Rec* Recession

In medial rectus muscle resection with an adjustable suture (Fig. 1B), full-thickness locking bite and half-thickness sutures on each edge of the muscle were placed 2 mm posterior to the desired resection point. The resected muscle was then suspended 2 mm posterior to the insertion site and secured using a single loop bowtie suture. This provided the space for advancement during the adjustment procedure if necessary. Recessed muscle was positioned with the originally desired surgical amount and only resected muscle was hanged by 2 mm. Because recessed muscle with hang back technique has enough place to advance if necessary when performing the adjustment surgery, but, resected muscle does not.

Adjustment procedures were performed 24 h after the initial surgery. After measuring the angle of deviation at distance and near fixation, the rectus muscle with an adjustable suture was fine-tuned for targeting slight overcorrection, from ≥ 5 prism diopters (PD) esotropia to < 15 PD esotropia. A single-loop half bowtie was untied under topical paracaine anesthesia, and the muscle was adjusted so that each pole of the muscle was positioned to the same length. After the rectus muscle was appropriately adjusted, a permanent square knot was tied. The additional traction suture and bolster suture were removed after the adjustment procedure was completed. Finally, conjunctival suturing was performed.

### Data evaluation

Enrolled patients were divided into two groups: those in whom the lateral muscle was recessed using adjustable suture (LR-Adj group), and those in whom the medial rectus muscle was resected using adjustable suture (MR-Adj group). Clinical factors, including sex, age at surgery, refractive errors, preoperative stereoacuity, angle of preoperative exodeviation, and type of surgical procedure, were analyzed. To evaluate the effectiveness of the surgical techniques, surgical success rate, angle of postoperative exodeviation, and amount of exodrift between the two groups were compared.

After the adjustment, horizontal deviation was measured 1 week, 1 month, 4 months, 10 months, and 18 months postoperatively. The pre- and postoperative angles of horizontal deviation were evaluated using the alternate prism cover test at distance (6 m) and near (33 cm) with spectacle correction in patients wearing glasses. All patients had cycloplegic refraction before surgery. Refractive errors were measured 1 h after instillation of 1% cyclopentolate, three times at 5 min intervals, and the results were converted to spherical equivalents. Stereoacuity was measured using two documents: Stereo Fly Stereotest (Stereo Optical Co., Chicago, Illinois, USA); and Frisby Davis Distance (FD2, Stereotest, Sheffield, UK).

Surgical success was defined as horizontal deviation < 5 PD esodeviation and < 10 PD exodeviation on distance measurement at 1.5 years postoperatively. Anisometropia was defined as > 1.5 D of the spherical equivalent and amblyopia was defined as an interocular difference in visual acuity of 2 lines or more.

### Statistical analyses

Data were analyzed using SPSS version 25.0 (IBM Corporation, Armonk, NY, USA). Comparison between the two groups were performed using the independent *t* test and chi-squared test. Differences with a p-value of < 0.05 were considered statistically significant and the values were reduced with Bonferroni correction when necessary.

## Results

Forty patients (14 male, 26 female) fulfilled the inclusion criteria for this study. The mean (± SD) follow-up period was 5.6 ± 4.3 years (range, 1.5–17 years) and the mean age at surgery was 31.6 ± 16.3 years (range, 9–66 years). Before strabismus surgery, the mean angle of exodeviation was 36.0 ± 13.0 PD (range, 18–65 PD) at distance measurement and 38.9 ± 13.0 PD (range, 18–70 PD) at near measurement. The mean refractive error was -2.3 ± 2.8 D in the right eye and -2.3 ± 3.3 D in the left eye.

Of the 40 patients, 21 comprised the LR-Adj group and 19 comprised the MR-Adj group. The preoperative characteristics of the two groups are summarized in Table 2. There were no statistical differences in sex, age at surgery, presence of amblyopia or anisometropia, mean refractive errors, preoperative stereoacuity, and angles of exodeviation between the two groups. There was no case of consecutive esotropia > 5 PD on the last follow-up in either group.

**Table 2:** Comparison of preoperative characteristics between LR-Adj group and MR-Adj group

	**LR-Adj group**	**MR-Adj group**	***P*—value**
Enrolled patient (n)	21	19	
Gender (male: female)	6: 15	8: 11	0.370 ^a^
Mean age at surgery (yr)	29.4 ± 17.2	34.1 ± 15.3	0.366 ^b^
Duration of follow-up (yr)	7.5 ± 4.7	3.4 ± 2.4	0.002 ^b^
Amblyopia (n)	2 (9.5%)	1 (5.3%)	0.538 ^a^
Anisometropia (n)	4 (19.0%)	4 (21.1%)	0.592 ^a^
Mean refractive errors (diopters of spherical equivalents, D)			
Right eye	-2.5 ± 2.6	-2.0 ± 3.2	0.601 ^b^
Left eye	-3.0 ± 3.2	-1.5 ± 3.3	0.147 ^b^
Angle of preoperative exodeviation (PD, range)			
Distance, mean	32.4 ± 12.3 (18 to 60)	39.8 ± 12.8 (20 to 65)	0.070 ^b^
Near, mean	35.4 ± 12.5 (18 to 65)	42.7 ± 12.7 (25 to 70)	0.073 ^b^
Stereoacuity			
Stereo Fly Stereotest ≤ 100 arcsec (n)	11/12 (91.7%)	9/12 (75.0%)	0.605 ^a^
Frisby Davis Distance (FD2) test (arcsec)	28.9 ± 13.4	33.5 ± 47.0	0.780 ^b^
Types of surgery			
Unilateral LR Rec	2	-	
Bilateral LR Rec	8	-	
Unilateral R and R	9	15	
Unilateral R and R with LR rec on the other eye	2	4	

All measurement data are presented as means ± standard deviations. ^a^ Chi square test; ^b^ Independent t test

*LR-Adj group* Adjustment on recessed lateral rectus group, *MR-Adj group* Adjustment on resected medial rectus group, *R and R* Lateral rectus recession and medial rectus resection, *LR* Lateral rectus, *MR* Medial rectus

**Fig. 2:**
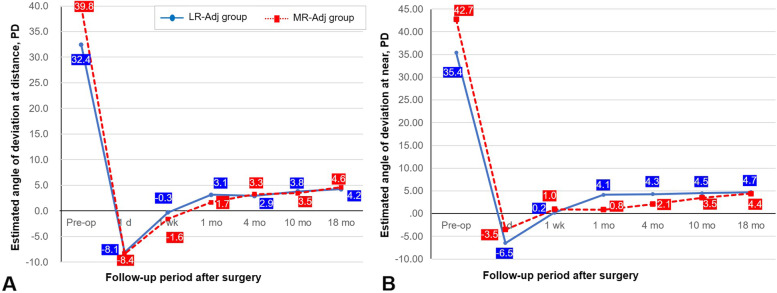
Changes in horizontal deviation after surgery. In the angle of deviation, the plus means the angle of exodeviation, and the minus means the angle of esodeviation. There was no significant difference in the angle of deviation between the two groups by time point. LR-Adj group = adjustment on recessed lateral rectus group, MR-Adj group = adjustment on resected medial rectus group, PD = prism diopter

Changes in horizontal deviation after surgery in the two groups are illustrated in Fig. 2. The adjustment procedure was performed with the patients under topical anesthesia 1 day after strabismus surgery in both groups. The mean esodeviation at distance measurement (Fig. 2A) immediately after adjustment was 8.1 ± 5.4 PD in the LR-Adj group and 8.4 ± 4.7 PD in the MR-Adj group (*P* = 0.843 [independent t test]). During follow-up, exodrift had occurred in both groups. The mean exodeviation at distance measurement 18 months postoperatively was 4.2 ± 7.6 PD in the LR-Adj group and 4.6 ± 3.9 PD in the MR-Adj group (*P* = 0.840 [independent *t* test]). Horizontal deviation changes observed at near measurement exhibited patterns similar to those of distance measurement (Fig. 2B). There was no significant difference in the angle of deviation between the two groups at each time point.

A comparison of the amount of exodrift from 1 day to 1 week, 1 month, 4 months, 10 months, and 18 months postoperatively is presented in Table 3. The amount of exodrift at distance measurement appeared to be larger in the LR-Adj group; however, the difference was not statistically significant. The amount of exodrift at near measurement from 1 day to 1 month postoperatively was significantly smaller in the MR-Adj group (4.0 ± 6.7 PD) than that in the LR-Adj group (10.4 ± 9.9 PD) (*P* = 0.01 [independent t test with Bonferroni correction]). However, there was no significant difference between the two groups at the last follow-up. Surgical success rates according to exodeviation at 18 months postoperatively were 81.0% in the LR-Adj group and 84.2% in the MR-Adj group (Table 4); however, the difference was not statistically significant (*P* = 0.559). Recurrence rates were 19.0% and 15.8%, respectively. There was no overcorrected case > 5 PD esotropia in either group.

**Table 3:** Amounts of exodrift at distance and near after surgery based on the postoperative 1 day

	**LR-Adj group**	**MR-Adj group**	***P*—value **^**a**^
Angle measurement at distance (PD)			
1 day to 1 week	7.8 ± 6.3	6.8 ± 4.7	0.590
1 day to 1 month	11.0 ± 7.6	10.1 ± 6.2	0.634
1 day to 4 months	11.2 ± 8.4	11.6 ± 6.4	0.762
1 day to 10 months	11.9 ± 9.1	11.8 ± 6.4	0.995
1 day to 18 months	12.3 ± 8.8	13.9 ± 6.1	0.770
Angle measurement at near (PD)			
1 day to 1 week	6.7 ± 7.8	4.0 ± 5.5	0.417
1 day to 1 month	10.4 ± 9.9	4.0 ± 6.7	0.010*
1 day to 4 months	10.8 ± 8.9	6.1 ± 6.8	0.069
1 day to 10 months	11.0 ± 9.4	7.0 ± 8.1	0.163
1 day to 18 months	11.2 ± 8.7	7.9 ± 7.7	0.221

All measurement data are presented as means ± standard deviations

^a^ Independent t test with Bonferroni correction

Significant differences are denoted by asterisk (*)

*LR-Adj group* Adjustment on recessed lateral rectus group, *MR-Adj group* Adjustment on resected medial rectus group, *PD* Prism diopter

**Table 4:** Surgical outcomes at 1.5 years postoperatively between two groups

	**LR-Adj group**	**MR-Adj group**	***P* – value **^**a**^
Surgical success, n (%)	17 (81.0)	16 (84.2)	0.559
Recurrence, n (%)	4 (19.0)	3 (15.8)	

^a^ Fisher’s exact test

*LR-Adj group* Adjustment on recessed lateral rectus group, *MR-Adj group* Adjustment on resected medial rectus group, *R and R* Lateral rectus recession and medial rectus resection, *LR* Lateral rectus, *MR* Medial rectus

## Discussion

In this study, postoperative changes in patients with intermittent exotropia who underwent adjustment surgery were evaluated. The mean final horizontal deviation and the surgical success rate were not different between the recessed muscle adjustable group and resected muscle adjustable group.

Since the popularization of the adjustment surgery in the 1970s [2, 10], many studies have addressed adjustment and focused on introducing different adjustment techniques [3, 11, 12] or demonstrating the effectiveness of the adjustment technique itself by comparing surgical results between adjustment and no adjustment groups [13, 14]. In addition, some studies focused specifically on conditions such as adjustment to thyroid-associated strabismus [15] or all types of strabismus including exotropia, esotropia, and vertical strabismus [9, 16]. As strabismus surgery for exotropia, lateral rectus recession or medial rectus resection can be performed. Due to the lack of extensive research evaluating surgical results of adjustment between the two muscles, the present study is meaningful because it compared the longitudinal clinical course after adjustment of the lateral rectus or medial rectus.

To eliminate the effect of several factors that may affect postoperative exodrift, only patients with basic type intermittent exotropia were enrolled. Patients with other types of horizontal strabismus, such as restrictive strabismus in thyroid- associated ophthalmopathy, and paralytic strabismus, such as cranial nerve palsy, were excluded. Although the number of enrolled patients is low due to the strict exclusion criteria, it is a homogenous cohort of basic type of intermittent exotropia.

The present study identified no statistically significant differences in postoperative horizontal deviation at defined time points and final surgical success rates between the two groups. The success rates of adjustment surgery in our study—81.0% in the LR-Adj group and 84.2% in the MR-Adj group—are comparable with success rates of reported in previous investigations,73.3–87.5% [8, 17–19].

Interestingly, we found significantly small amounts of postoperative exodrift at 1 month after surgery in the MR-Adj group, which may have several explanations. First, it is possible that the lateral rectus muscle that was recessed using the hang back technique migrated anteriorly from the intended position, which could have resulted in more exodrift after surgery in the LR-Adj group. Furthermore, the resected muscle in the MR-Adj group may have had more tension and could be more stable in the intended position than the hang back recessed muscle in the LR-Adj group. In addition, the smaller exodrifts in the MR-Adj group at 1 month postoperatively were more prominent in near deviation. This could be due to the action of the medial rectus muscle, which is preferentially influenced at near fixation [20]. Meanwhile, there were no significant differences between the groups in terms of final exodeviation and surgical success rate. Although the exact mechanism explaining why the short-term or long-term outcomes were different remains unclear, it appears to be that the stability of adjusted resected muscle was better and tension of the resected muscle strongly resists exodrift in the direction opposite to the exodrift. Ultimately, however, this effect does not last long. It is possible that the long-term result is influenced by other factors, such as patient fusional capacity rather than the stability and tension of the muscle itself. In addition, this difference is also can be due to the fact that the recess resect procedure itself behaves differently from the bilateral recess procedure regardless of the adjustment procedure.

There is considerable literature addressing resected muscle adjustment [8, 9, 12]. Hong et al. [8] reported that they resected the rectus muscle and adjusted it in patients with sensory exotropia. The authors noted that this technique could prevent over- or under-correction and result in favorable surgical outcomes. In addition, Ogut et al. [9] described that they primarily performed the adjustment surgery on the resected muscle because recession of the muscle using adjustment surgery may be associated with undesired vertical deviation with instability.

Similarly, we also suggest that our method of resected muscle adjustment offers potential advantages in the surgical technique. First, the resected muscle body is positioned near the insertion site; this provides better exposure during the adjustment procedure. Each pole of the fine-tuned muscle can be easily checked if positioned to the same length. Second, the resected muscle adjustment does not require far hang back as in recessed muscle adjustment. Thus, the stability of the muscle position is improved and, although rare, the risk for the hang-backed recessed muscle to come forward to the insertion site postoperatively is reduced. Furthermore, the surgical success rate at 1 year was not low compared with that of conventional recessed muscle adjustment.

There are certain limitations to our study. First, anatomical differences in the medial rectus and lateral rectus muscles were not considered. Anatomically, there are several differences between these two recti muscles, including length and intramuscular nerve distribution [21]. Because it is unclear whether these differences affect eye position changes after strabismus surgery, this limitation could not be controlled. Second, this retrospective study did not unify the type of surgery in patients with the same amount of exodeviation (Table 1); as such, differences in surgery type may have biased long-term results. Although there was no significant difference in preoperative exodeviation between the LR-Adj and MR-Adj groups, the combination of two types of surgery—BLR (i.e., bilateral lateral rectus recession) and R&R (i.e., recession and resection)—can affect the outcomes of adjustable strabismus surgery, which was an additional limitation of this study. Thus, a better study design would have been to include patients with only a recess-resect procedure and compare whether keeping the lateral rectus on adjustment or medial rectus on adjustment had any effect on the outcome.

## Conclusions

Adjustment of the resected medial rectus muscle had comparable surgical outcomes with the adjustment of the recessed lateral rectus muscle in the patients with basic type intermittent exotropia. Moreover, resected medical rectus adjustment yielded smaller exodrift in the early postoperative period and it may mean that resected muscle is more stable in the intended position than the hang back recessed muscle. Due to the limitation of the small sample size enrolled in this study, a prospective, randomized, comparative clinical trial with a larger number of cases will be helpful to confirm the effectiveness of resected muscle adjustment.

## Data Availability

The datasets used and/or analyzed during the current study are available from the corresponding author on reasonable request.

## Abbreviations

D: Diopters
LR: Lateral rectus
LR-Adj group: Adjustment on recessed lateral rectus group
MR-Adj group: Adjustment on resected medial rectus group
PD: Prism diopters
R and R: Lateral rectus recession and medial rectus resection
Rec: Recession
